# The Antihistamine Drugs Carbinoxamine Maleate and Chlorpheniramine Maleate Exhibit Potent Antiviral Activity Against a Broad Spectrum of Influenza Viruses

**DOI:** 10.3389/fmicb.2018.02643

**Published:** 2018-11-06

**Authors:** Wei Xu, Shuai Xia, Jing Pu, Qian Wang, Peiyu Li, Lu Lu, Shibo Jiang

**Affiliations:** ^1^Shanghai Public Health Clinical Center and School of Basic Medical Sciences, Key Laboratory of Medical Molecular Virology of MOE/MOH, Fudan University, Shanghai, China; ^2^Lindsley F. Kimball Research Institute, New York Blood Center, New York, NY, United States

**Keywords:** antihistamine, carbinoxamine, chlorpheniramine, influenza virus, viral entry

## Abstract

Influenza A viruses (IAV) comprise some of the most common infectious pathogens in humans, and they cause significant mortality and morbidity in immunocompromised people as well as children and the elderly. After screening an FDA-approved drug library containing 1280 compounds by cytopathic effect (CPE) reduction assay using the Cell Counting Kit-8, we found two antihistamines, carbinoxamine maleate (CAM) and S-(+)-chlorpheniramine maleate (SCM) with potent antiviral activity against A/Shanghai/4664T/2013(H7N9) infection with IC_50_ (half-maximal inhibitory concentration) of 3.56 and 11.84 μM, respectively. Further studies showed that CAM and SCM could also inhibit infection by other influenza A viruses, including A/Shanghai/37T/2009(H1N1), A/Puerto Rico/8/1934(H1N1), A/Guizhou/54/1989(H3N2), and one influenza B virus, B/Shanghai/2017(BY). Mice were challenged intranasally with A/H7N9/4664T/2013 (H7N9) virus and intraperitoneally injected with CAM (10 mg/kg per day) or SCM (1 mg/kg per day) for 5 days. CAM or SCM (10 mg/kg per day) were fully protected against challenge with A/Shanghai/4664T/2013(H7N9). The results from mechanistic studies indicate that both could inhibit influenza virus infection by blocking viral entry into the target cell, the early stage of virus life cycle. However, CAM and SCM neither blocked virus attachment, characteristic of HA activity, nor virus release, characteristic of NA activity. Such data suggest that these two compounds may interfere with the endocytosis process. Thus, we have identified two FDA-approved antihistamine drugs, CAM and SCM, which can be repurposed for inhibiting infection by divergent influenza A strains and one influenza B strain with potential to be used for treatment and prevention of influenza virus infection.

## Introduction

Influenza is an acute respiratory illness caused by influenza virus infection, resulting in more than 5 million severe cases and 650,000 deaths worldwide every year^1^. Although most influenza virus infections are self-limiting, they can sometimes be associated with severe, even fatal, disease outcomes, such as pneumonia and congestive heart failure (Rockman et al., 2017). Influenza viruses are classified into types A, B, and C. Influenza A viruses (IAVs) have undergone frequent changes through mutation or reassortment, resulting in the appearance of new strains that can cause an epidemic or pandemic^2^. For example, an outbreak of novel avian IAV influenza A virus (H7N9) infection was reported in China in April of 2013. This has caused at least 1,564 deaths during the 5 major epidemic waves that followed (Qiu et al., 2013; Zhu et al., 2016). In the past few years, several highly pathogenic avian influenza (HPAI) viruses, including H5N8, H5N6 and H10N8, were identified, raising global public health concerns (Chen et al., 2014; Xu et al., 2017; Zhang et al., 2017). This calls for rapid identification of antiviral agents to prevent and treat divergent influenza virus infection.

Currently, two major classes of antiviral drugs are available for the treatment of influenza, including M2 ion channel protein inhibitors (e.g., amantadine and rimantadine) and influenza neuraminidase inhibitors (NAIs, e.g., oseltamivir, zanamivir, and peramivir) (Ong and Hayden, 2007). M2 ion channel protein inhibitors are only active against IAVs, yet 95% of IAV strains are resistant to amantadine (Yen et al., 2005; Salter et al., 2011). Although NAIs like oseltamivir are effective against both influenza A and B viruses (Baranovich et al., 2014), a large variety of influenza virus strains have become resistant to oseltamivir because of its extensive clinical application (Matsuzaki et al., 2010; Pizzorno et al., 2014; Marjuki et al., 2015). Lack of effective treatments for influenza and the continuous emergence of drug resistance highlight the urgency of developing new drugs for treatment of influenza virus infection.

Drug discovery is extremely expensive and time-consuming. It generally takes 10–15 years and billions of dollars to reach clinical trials (DiMasi et al., 2003). Thus, for rapidly spreading and emerging infectious diseases, an alternative approach to identify new antiviral agents lies in the repurposing of existing drugs used in clinics since these FDA-approved drugs have already been proven safe in many previous clinical trials (Chong and Sullivan, 2007).

Using this strategy, some FDA-approved drugs for non-infectious diseases have shown efficacy against infection caused by such emerging viruses as Ebola virus and Zika viruses (Xu et al., 2016). Following the logic of this investigative path, we screened an FDA-approved drug library containing 1,280 compounds and found two antihistamines, carbinoxamine maleate (CAM) and S-(+)-chlorpheniramine maleate (SCM), with potent antiviral activity against A/Shanghai/4664T/2013(H7N9) infection with IC_50_ (half-maximal inhibitory concentration) at low μM level.

CAM (brand names: Palgic, Histex PD, Karbinal ER, Carboxine, Ryvent) and SCM (brand names: Chlor-Trimeton, Allergy Relief, Chlorphen) are H1-receptor blocking agent-based antihistamine drugs approved by the U.S. FDA in 1950s and 1982 for treatment of hay fever, rhinitis, urticaria, and asthma (Halpern et al., 1965; Falagas and Kasiakou, 2005; Bergen et al., 2006; Li et al., 2006; Nation et al., 2016). At present, CAM- and SCM-containing formulations are approved only for adults or children ages 2 or older. In this study, we evaluated their antiviral activity against divergent influenza A and B viruses in vitro and in vivo, specifically IAV H7N9 infection in mice, and investigated their potential mechanism of action.

## Materials and Methods

### Chemical Library and Compounds

The FDA-approved drug library containing 1,280 compounds was obtained from MicroSource Discovery Systems, Inc. (Gaylordsville, CT, United States). Compounds were solubilized in dimethyl sulfoxide (DMSO) at 10 mM and stored in 96-well PCR Tubes at -80°C. Carbinoxamine maleate salt (CAM) and S-(+)-Chlorpheniramine maleate salt (SCM) were purchased from Sigma-Aldrich (St. Louis, MO, United States). Favipiravir (T-705) and Oseltamivir phosphate were purchased from TargetMol Company (Boston, MA, United States).

### Cells, Viruses and Plasmids

Madin-Darby canine kidney (MDCK) cells, 293T cells, U87 cells and Huh-7 cells were obtained from ATCC (Manassas, VA, United States). Cells were cultured in DMEM (Thermo Fisher Scientific, Waltham, MA, United States) containing 10% FBS (Biowest, France), penicillin 100 U/ml, and streptomycin 10 μg/ml. Influenza virus strains A/Shanghai/37T/2009(H1N1), A/Puerto Rico/8/1934(H1N1), A/Guizhou/54/1989(H3N2), A/Shanghai/4664T/2013(H7N9), and B/Shanghai/2017(BY) were obtained from the Shanghai Public Health Clinical Center (Shanghai, China). The experiments on H7N9 IAV were conducted in the BSL-3 Laboratory of Fudan University. Live viruses were propagated in MDCK cells. Virus titers were determined by a TCID_50_ endpoint dilution assay. Plasmid pNL4-3.Luc.R-E- was obtained from the NIH AIDS Reagent Program (Germantown, MD, United States).

### CCK-8 Assay for Measuring Cytotoxicity of the Compounds Tested

The cytotoxicity of a test compound was determined, as previously described (Furuta et al., 2002; Han et al., 2011) by measuring cell viability in the presence of various compound concentrations. Briefly, 100 μl MDCK cells (0.5 × 10^5^ cells/ml) were dispensed into wells of a 96-well plate and incubated at 37°C/5% CO_2_ for 24 h. The test compound at the serial dilution in 100 μL of DMEM without serum was added to the cells. After culture for 72 h, cell viability was measured using the Cell Counting Kit-8 (CCK-8), which was purchased from Dojindo Laboratory (Kumamoto, Japan), and using a spectrophotometer (Ultra 384, Tecan, NC, United States). The percent cytotoxicity (=100% – % cell viability) was calculated, and CC_50_ (half-maximal cytotoxic concentration) of the test compound was determined using the Calcusyn software program (Biosoft, Ferguson, MO, United States) (Chou and Talalay, 1984).

### Cytopathic Effect (CPE) Reduction Assay for Measuring Inhibitory Activity of a Compound on Cell Death Caused by Influenza Virus Infection

The inhibitory activity of a test compound against influenza virus infection-induced death of MDCK cells was measured by CPE reduction assay using CCK-8 as previously described (Choi et al., 2016; Wang et al., 2017; Jin et al., 2018; Liu et al., 2018). PBS containing no inhibitor and the anti-influenza virus agent, Favipiravir (T-705), were included as the negative and positive controls, respectively. Briefly, MDCK cells were plated in wells of a 96-well plate at a density of 10^4^ cells/well. After incubation at 37°C for 12 h, a mixture of a test compound (50 μL) at graded concentration in serum-free DMEM containing 2 μg/mL tosyl phenylalanyl chloromethyl ketone (TPCK)-Trypsin and 50 μL of a influenza virus (100 TCID_50_) was added to the monolayer cells, followed by an incubation at 37°C for 8 h. The supernatants were replaced with DMEM medium containing 2 μg/mL TPCK-Trypsin. After culture at 37°C for 72 h, the inhibitory activity of the compound on cytopathic effect induced by viral infection was measured using CCK-8 as described above. The IC_50_ values were calculated with the Calcusyn Program (Biosoft).

A time-of-addition assay was performed as previously described (Maddry et al., 2011). Briefly, the A/Shanghai/37T/2009(H1N1) at an MOI of 0.1 was added to the monolayer MDCK cells, followed by addition of a test compound at indicated concentration of 0, 0.5, 1, 2, 4, and 12 h post-infection (p.i.). The inhibitory activity of the compound on H1N1 IAV infection was detected by CPE reduction assay using CCK-8 as described above.

### Quantitative Reverse Transcription PCR (RT-qPCR) Assay for Measuring the Inhibitory Activity of a Test Compound on Expression of mRNA of Influenza a HA Gene

MDCK cells were seeded onto 12-well plates (2 × 10^5^/well) and cultured overnight. The cells were infected with approximately 100 pfu/ml of the influenza A/PR/8/34 (H1N1) virus for 1h in the absence or presence of CAM and SCM at the indicated concentration and then the medium was replaced with fresh DMEM containing 1.5 μg/ml TPCK-Trypsin. After incubation at 37°C for 12 h, total RNA was isolated from cells using Trizol Reagent (TransGen, China). The First-stranded cDNA was reverse by TransScript First-Strand cDNA Synthesis SuperMix (TransGen, China) according to the instructions. The qPCR assay was performed by using the TransStart Top Green qPCR SuperMix. The primer sequences used for the influenza A HA genes are 5′-TTCCCAAGATCCATCCGGCAA-3′ (forward) and 5′-CCTGCTCGAAGACAGCCACAACG-3′ (reverse); NP genes are 5′-GACCAGGAGTGGAGGAAACA-3′ (forward) and 5′-CGGCCATAATGGTCACTCTT-3′ (reverse); and the sequences for the GAPDH gene are 5′-AGGGCAATGCCAGCCCCAGCG-3′ (forward) and; 5′-AGGCGTCGGAGGGCCCCCTC-3′ (reverse).

### Indirect Immunofluorescence Assay for Measuring the Inhibitory Activity of a Test Compound on Expression of Influenza Virus Nucleoprotein (NP) in Virus-Infected Cells

MDCK cells (1 × 10^4^ cells/well) were seeded onto 96-well plates at 37°C under 5% CO_2_ for 8 h. The cells were washed twice with phosphate-buffered saline (PBS) and infected with 100 TCID_50_ of the influenza A/PR/8/34 (H1N1) virus in the presence or absence of CAM and SCM at the indicated concentration for 1 h. The supernatant was aspirated and covered with culture medium containing 1.5 μg/ml TPCK-Trypsin. After incubation at 37°C for 12 h, the cells were fixed with 4% paraformaldehyde for 10 min, then permeabilized with 0.1% Triton^TM^ X-100 for 10 min, blocked with 1% BSA for 1 h and labeled with a primary antibody against influenza virus A nucleoprotein (NP) (1:250 dilution, Santa Cruz, CA, United States) for 3 h. Goat anti-Mouse IgG (H + L) Highly Cross-Adsorbed Secondary Antibody Alexa Fluor^®^ 488 conjugate (diluted 1:250) was added at 37°C for 1 h. After further washing, the nuclei were stained with DAPI for 10 min. The cells were observed and photographed by using a fluorescence microscope.

### Plaque Reduction Assay for Measuring the Inhibitory Activity of a Test Compound on Plaque Formation Induced by Influenza Virus Infection

Briefly, MDCK cells were plated onto 12-well culture plates (1 × 10^6^ cells/well) and cultured overnight. MDCK cells were washed twice with PBS and incubated with approximately 100 pfu/ml of the influenza A/PR/8/34 (H1N1) virus in the presence or absence of CAM and SCM at the indicated concentration for 1 h. After incubation at 37°C for 1 h, the supernatant was aspirated. The cells were washed once with PBS and were covered with DMEM containing 1% low melting agarose and 1.5 μg/ml TPCK-trypsin. After 2–3 days post-infection, the cell monolayer was fixed with 4% formalin and stained with 1% crystal violet for 1 h and the plaque forming units were counted.

### Luminescence Assay for Detecting Inhibitory Activity of a Test Compound on Entry of the Pseudotyped H7N9 IAV, Nipah Virus and VSV Into Their Target Cells

Pseudotyped H7N9 IAV, Nipah virus, and vesicular stomatitis virus (VSV) were constructed, and their infectivity was evaluated as previously described (Qiu et al., 2013). For the pseudotyped H7N9 influenza virus 293T cells (5 × 10^5^ cells/well) were seeded to six-well plate the day before transfection, and co-transfected with 2.5 μg of pcDNA3.1-HA and NA of A/Shanghai/4664T/2013(H7N9), respectively, and 10 μg of lentivirus vector pNL4-3-Luc R-E- by using VigoFect reagents (Vigorous Biotechnology, Beijing, China). For the pseudotyped Nipah virus or VSV, 293T cells were co-transfected with Nipah G protein or VSV G protein, respectively, and plasmid encoding Env-defective, lentivirus vector pNL4-3-Luc R-E- by using VigoFect reagents. After incubation at 37°C for 6 h, cells were washed with PBS 3 times and cultured in DMEM containing 10% FBS. After culture at 37°C/5% CO_2_ for 2 days, supernatants containing the pseudoviruses were collected and filtered to remove cells and cell debris. The pseudoviruses were concentrated by ultracentrifugation at 25,000 rpm at 4°C for 3 h, and then their titers were determined using the Luciferase Assay System (Promega, Madison, WI, United States), following the manufacturer’s instructions. Aliquots of the pseudoviruses were stored at -80°C.

The inhibitory activity of a test compound on entry of H7N9, Nipah or VSV pseudovirus into their target cells was determined as described previously (Xia et al., 2018). Briefly, MDCK cells for H7N9 pseudovirus entry, Huh-7 cells for Nipah pseudovirus entry, and U87 cells for VSV pseudovirus entry were plated in wells of a 96-well plate at a density of 10^4^ cells each well, respectively. After incubation at 37°C for 12 h, a mixture of a test compound (50 μL) at a serial twofold dilution in serum-free DMEM and 50 μL of the corresponding pseudovirus was added to the corresponding cells. MDCK, Huh7 and U87 cells were infected by these control viruses, as described above, followed by incubation at 37°C for 12 h. The culture supernatants were replaced with free DMEM containing 2% FBS. After culture at 37°C for 48 h, the luciferase activity was measured using the Luciferase Assay System (Promega) with a microplate luminometer (Ultra 384, Tecan, NC, USA). The inhibitory activity of a test compound on entry of HIV-1 IIIB infection were determined as described previously (Su et al., 2017a,b). Series dilution of CAM and SCM were incubated with 50 μL 100 TCID_50_ of the HIV-1 at 37°C for 1 h and then 10^4^ MT-2 cells per well were added into the 96 well plate. After 12 h culture, the medium was replaced with fresh RPMI 1640 medium containing 10% FBS. After culture at 37°C for 4 days, the supernatant was mixed with 50 μL of 5% Triton X-100. The p24 antigen was detected by ELISA. The IC_50_ values were calculated with the Calcusyn Program (Biosoft).

### Neuraminidase (NA) Inhibition Assay

Neuraminidase inhibition assay was performed to investigate the influence of a test compound on the release of newly produced viral particles, as described previously (Shen et al., 2018). Briefly, 15 μL of A/Puerto Rico/8/1934(H1N1) virus solution were mixed with 5 μL of a test compound at graded concentrations in wells of a 96-well black plate, followed by incubation at 37°C for 30 min. Then, 30 μL of 20 μM MU-NANA 2′-(4-Methylumbelliferyl)-alpha-D-*N*-acetylneuraminic acid sodium salt hydrate (Sigma-Aldrich, United States), as the substrate dissolved in the MES (2-(*N*-Morpholino) ethanesulfonic acid buffer (32.5 mM MES and 4 mM CaCl_2_, pH 6.5), were added to each well, followed by incubation at 37°C for 1 h in the dark. Subsequently, 50 μL of 14 mM NaOH were added to terminate the enzymatic reaction. The fluorescence intensity of the product 4-methylumbelliferone was recorded using a microplate reader with excitation and emission wavelengths of 340 and 440 nm, respectively. The IC_50_ values were calculated with the Calcusyn Program (Biosoft).

### Haemagglutination Inhibition (HI) Assay

The HI assay was performed to measure the inhibitory activity of a test compound on attachment of an influenza virus to red blood cells (RBC) through the interaction between HA on virus and receptor on RBC, as described previously (Shen et al., 2018). A/Shanghai/37T 2009(H1N1) virus or H7N9 pseudovirus at the concentration of 4 HA units were prepared. Compounds with twofold serial dilution with PBS were mixed with equal volume of influenza virus solution (4 HAU per 25 μL). After incubation at room temperature for 1 h, 50 μL of 1% chicken RBC in PBS were added to the mixture. HI was observed and recorded after 1h at room temperature.

### Animal Experiment for Evaluating the *in vivo* Inhibitory Activity of a Test Compound on Influenza Virus in Mice

The animal experimental procedure was carried out according to ethical guidelines and approval by Shanghai Public Health Clinical Center Animal Welfare and Ethics Committee (2017-A046-01). The animal studies with infectious H7N9 IAV were conducted in a Biosafety Level 3 facility of Fudan University with Institutional Biosafety Committee approval.

Female C57BL/6 mice (4–8 weeks old, specific-pathogen-free) purchased from Shanghai Lingchang BioTech co., Ltd. (Shanghai, China) and randomly divided into 7 groups (*n* = 12): mock-infection group, PBS group, CAM high dose group (10 mg/kg/day), CAM low dose group (1 mg/kg/day), SCM high dose group (10 mg/kg/day), SCM low dose group (1 mg/kg/day) and OSE group (1 mg/kg/day). Except for mice in the mock-infection control group that received 20 μL DMEM and 20 μL PBS, mice in the treatment groups were anesthetized by intraperitoneal injection of pelltobarbitalum natricum (He et al., 2016; Yamayoshi et al., 2017) and challenged intranasally with A/H7N9/4664T/2013 (H7N9) (3.1 × 10^7^ TCID_50_ in 20 μL DMEM), following by i.p. application of 20 μL PBS (for PBS control group) or a compound in 20 μL PBS (for each compound-treatment group). On the 6th day post infection, 3 mice in each group were euthanized with CO_2_ inhalation and their left lungs were removed. The lungs were then immersed in TRIzol^TM^ reagent and frozen at -80°C. Total RNA was isolated from lung homogenates. The levels of viral RNA were determined by two-step RT-qPCR kit (TransGen, China). The right lungs were placed in 4% paraformaldehyde at 4°C for hematoxylin-eosin (H&E) staining and histological analysis. The body weight of the mice was monitored daily for 14 days after infection. Survival curves were generated according to the Kaplan–Meier method and the differences was calculated by Log-rank (Mantel-Cox) Test (GraphPad Prism 6.0).

### Histopathological Analysis

The formalin-fixed paraffin-embedded tissues were cut into 4 μm sections and stained with hematoxylin and eosin (H&E). Observation and description were made after scanning in the panoramic scanner (3D HISTECH Pannoramic MIDI, Hungary). Blinded sections were examined by two independent pathologists and scored for the following indicative parameters: (A) alveolar congestion, (B) hemorrhage, (C) Neutrophilic infiltration, (D) hyaline membrane formation, (E) thickness of the alveolar wall (0 = non-observed damage, 1 = mild damage, 2 = moderate damage, 3 = severe damage, and 4 = maximal damage). Ten non-overlapping microscopic images were obtained from each tissue sample with 40x objective for observation.

### Immunohistochemical Assay

Immunohistochemical staining of viral NP was performed. The right lung of each mouse was fixed in 10% buffered formalin solution for 7 days and embedded in paraffin. The embedded tissue was cut into 4 μm sections and stained with a primary mouse polyclonal antibody against NP of influenza A virus (Santa Cruz Biotechnology, Santa Cruz, CA, United States) at 1:1000 and a secondary HRP-labeled goat anti-mouse IgG antibody (Thermo Fisher Scientific) at 1:200. The integrated optical density (IOD) value was measured by Image-Pro Plus 6.0 software (Media Cybernetics, Inc., Rockville, MD, United States). The images were captured under an optical microscope (×200). The same brown and yellow color was selected as the unified standard to judge the positivity of all images. The IOD values in three randomly selected views were determined. The mean IOD value was considered as the relative expression levels of viral NP.

## Results

### CAM and SCM Exhibited Potent and Broad Antiviral Activity Against Influenza Virus Infection in MDCK Cells, as well as Low Cytotoxicity

**FIGURE 1:**
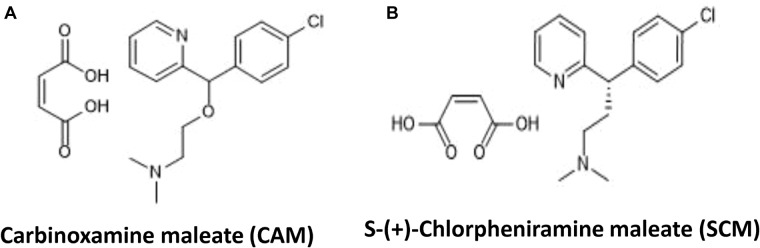
**(A,B)** Chemical structures of carbinoxamine and S-(+)-Chlorpheniramine maleate. The chemical structure of compounds were download from ChemACX.Com and edited by ChemDraw Professional 16.0 software.

**FIGURE 2:**
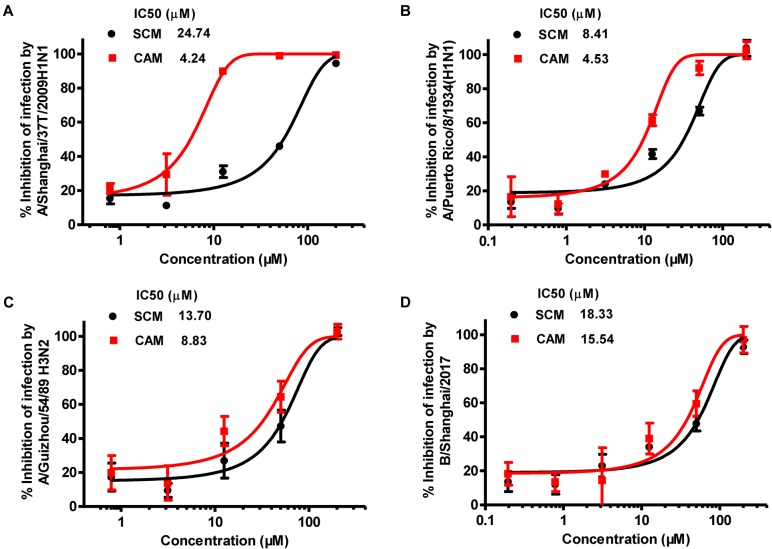
Inhibition of CAM and SCM on influenza virus infection in vitro. The IC_50_ values of CAM and SCM were measured for their inhibition of infection by: **(A)** A/Shanghai/37T/2009(H1N1); **(B)** A/Puerto Rico/8/1934(H1N1); **(C)**: A/Guizhou/54/1989(H3N2); **(D)** B/Shanghai/2017(BY) in MDCK cells at 100 TCID_50_ in the presence of increasing concentrations of the compounds. The inhibitory activity of CAM and SCM on cytopathic effect (CPE) caused by influenza virus infection was determined at 72 h post infection by CPE reduction assay using CCK-8. Data are expressed as mean ± standard error (SE) of triplicate assays, and the experiment was repeated at least twice.

To identify chemical compounds with broad antiviral activity against divergent influenza viruses, we previously screened a FDA-approved drug library that includes 1280 FDA-approved drugs (Gaylordsville, CT, United States) against H7N9 influenza virus infection by CPE reduction assay using CCK-8 for identifying compounds with inhibitory activity against influenza virus infection-induced cell death at the concentrations of the compounds below their CC_50_. We found two antihistamines, carbinoxamine maleate (CAM) and S-(+)-chlorpheniramine maleate (SCM) (Figure 1), with potent antiviral activity against infection of A/Shanghai/4664T/2013(H7N9) with IC_50_ of 3.56 and 11.84 μM, respectively (Table 1). We then further evaluated their inhibitory activity against three different influenza A virus subtypes isolated from humans, including A/Shanghai/37T/2009(H1N1), A/Puerto Rico/8/1934(H1N1), and A/Guizhou/54/1989(H3N2), as well as one influenza B virus, B/Shanghai/2017(BY). The IC_50_ values of the compounds were determined by CPE reduction assay using CCK-8. Both CAM and SCM exhibited effective inhibitory activity against infection by these influenza viruses in a dose-dependent manner with IC_50_ values ranging from 4.2 to 24.7 μM (Figure 2 and Table 1). Favipiravir (T-705) exhibited potent antiviral activity against A/Guizhou/54/89(H3N2) infection with IC_50_ of 0.746 μM (Supplementary Figure S4). We then performed a plaque reduction assay and found that CAM and SCM were effective in reducing plaque formation caused by A/Shanghai/37T/2009(H1N1) and A/Guizhou/54/1989(H3N2) infection with IC_50_ in a range of 2.7–14.5 μM (Supplementary Table S4).

**Table 1:** Anti-influenza virus activity of CAM and SCM.

		Compound	
		CAM	SCM
	CC50 (μM)	297.30 ± 11.11	285.30 ± 10.89
Influenza A	IC50 (μM)	4.53 ± 1.51	8.41 ± 2.18
[A/Puerto	SI	65.73	33.92
Rico/8/1934(H1N1)]	
Influenza A	IC50 (μM)	8.83 ± 2.94	13.70 ± 1.74
[A/Guizhou/54/	SI	33.67	20.82
1989(H3N2)]	
Influenza A	IC50 (μM)	4.24 ± 0.39	24.74 ± 2.57
[A/Shanghai/37T/	SI	70.11	11.53
2009(H1N1)]	
Influenza B	IC50 (μM)	15.54 ± 6.40	18.33 ± 2.33
[B/Shanghai/2017(BY)]	SI	19.13	15.56
Influenza A	IC50 (μM)	3.56 ± 1.09	11.84 ± 2.64
[A/H7N9/4664T/	SI	83.51	24.09
2013 (H7N9)]	

*CC_*50*_, half-maximal cytotoxic concentration; IC_*50*_, half-maximal inhibitory concentration; SI, selectivity index (=CC_*50*_/IC_*50*_)*.

The *in vitro* antiviral efficacy of CAM and SCM was also evaluated by using RT-qPCR assay for assessing the levels of viral gene expression. The results showed that CAM and SCM could downregulate viral RNA synthesis in a dose-dependent manner, compared with the PBS control (Supplementary Figures S1A,B). The expression of the viral NP in the MDCK cells infected with the influenza A/PR/8/341934 (H1N1) was analyzed by indirect immunofluorescence staining assay. As shown in Supplementary Figure S1C, addition of CAM and SCM at 10 μM resulted in marked reduction in the number of fluorescent-staining cells, compared to the PBS control. All these results indicate that CAM and SCM possess relatively broad-spectrum antiviral activities against divergent influenza A strains and one influenza B strain.

**FIGURE 3:**
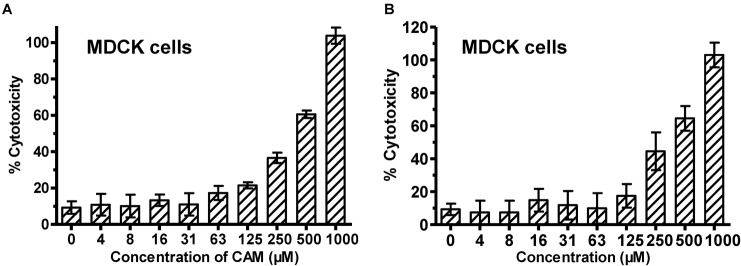
Cytotoxicity of CAM **(A)** and SCM **(B)** to MDCK cells. MDCK cells were incubated with the test compounds at graded concentration for 72 h before the cell viability was measured by CCK-8 assay. The samples were tested in triplicate. CC_50_ was calculated as described in the “Materials and Methods.” Data are expressed as mean ± standard error (SE) of triplicate assays, and the experiment was repeated at least twice.

We then determined the cytotoxicity of these compounds to MDCK cells using CCK-8 assay and found that both CAM and SCM exhibited low cytotoxicity to MDCK cells with CC_50_ values of 297 ± 11 and 285 ± 11 μM, respectively (Figure 3 and Table 1). Accordingly, the selectivity index (SI = CC_50_/IC_50_) of CAM and SCM were in the range of 12 – 84 (Table 1). These data suggest that both CAM and SCM have effective *in vitro* anti-influenza virus activity with low cytotoxicity.

### CAM and SCM Protect Against H7N9 Influenza Virus Infection in Mice

**FIGURE 4:**
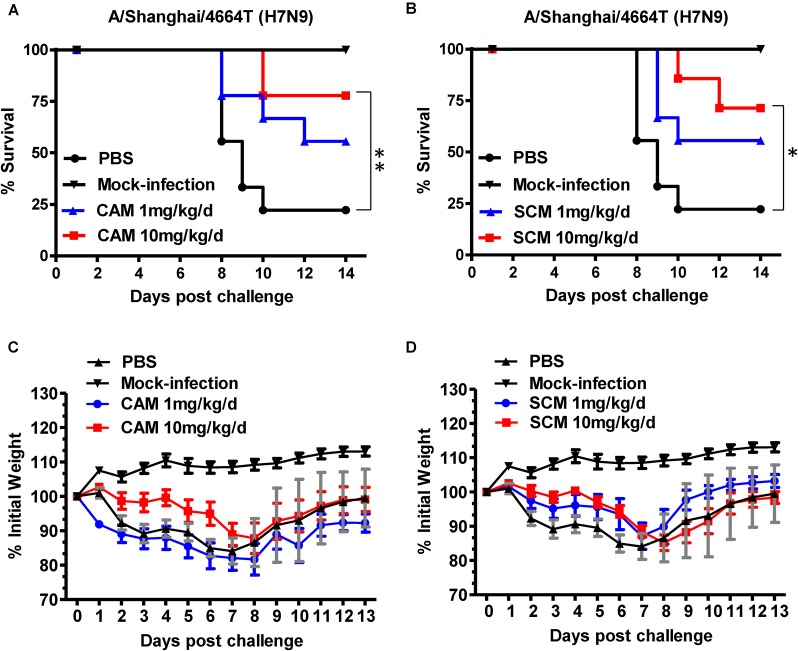
Evaluation of CAM and SCM for efficacy against a highly pathogenic influenza A/4664T/2013 (H7N9) virus infection in C57BL/6 mice. **(A)** Survival of H7N9 infected mice treated with CAM (1 mg/kg/day or 10 mg/kg/day); **(B)** Survival of H7N9 infected mice treated with SCM (1 mg/kg/day or 10 mg/kg/day); Changes in body weight of virus-infected mice treated with CAM **(C)** or SCM **(D)**. C57BL/6 mice anesthetized with pelltobarbitalum natricum (10 mg/ml) were infected with 3.1 × 10^7^ TCID_50_ of the influenza A virus strain (A/4664T/2013 H7N9). CAM and SCM (1 mg/kg/day or 10 mg/kg/day) was intraperitoneally administered to C57BL/6 mice 1 h post infection once daily for 5 days. PBS and oseltamivir phosphate (OSE, 1 mg/kg/day) were used as a negative and positive controls, respectively. Survival curves were generated according to the Kaplan–Meier method and the differences were analyzed by Log-rank (Mantel-Cox) Test (GraphPad Prism 6.0). The data were obtained from a single experiment. ^∗^Indicates a statistically significant difference (*P* < 0.05) and ^∗∗^represents significant difference at *P* < 0.01 in survival rate between treated group and the PBS group.

To evaluate the *in vivo* efficacy of CAM and SCM for protection against H7N9 IAV infection, C57BL/6 mice were inoculated with 3.1 × 10^7^ TCID_50_ A/Shanghai/4664T/2013(H7N9) and intraperitoneally injected with CAM and SCM at both high dose (10 mg/kg/day) and low dose (1 mg/kg/day), respectively, daily for 5 days beginning 1 h after infection. Mice in the positive and negative control groups were treated with OSE, a neuraminidase inhibitor (Tamiflu, 1 mg/kg/day), or PBS, respectively. As shown in Figure 4, the mice intraperitoneally injected with CAM and SCM were protected from challenge with H7N9 IAV in a dose-dependent manner. The survival rates of the mice treated with CAM or SCM at high dose (10 mg/kg/day) for 5 days were 77.7 and 71.4%, respectively, while those of mice treated with OSE or PBS were 66.6 and 22.2%, respectively.

We then examined the lung tissues of H7N9- or mock-infected mice treated or untreated with CAM or SCM for the virus gene (mRNA for HA) expression by using a two-step RT-qPCR kit. The results showed that both CAM and SCM could significantly downregulate the expression of viral gene in the mouse lung tissues (Supplementary Figure S2). The viral NP mRNA levels in the H7N9-infected mice treated with OSE, high dose of CAM, high and low doses of SCM, and the mock-infected mice were significantly lower than that in the H7N9-infected mice without treatment (PBS control) (Supplementary Figure S2). These results suggest that like OSE, both CAM and SCM are highly effective in inhibiting H7N9 replication in the mouse lung tissues.

**FIGURE 5:**
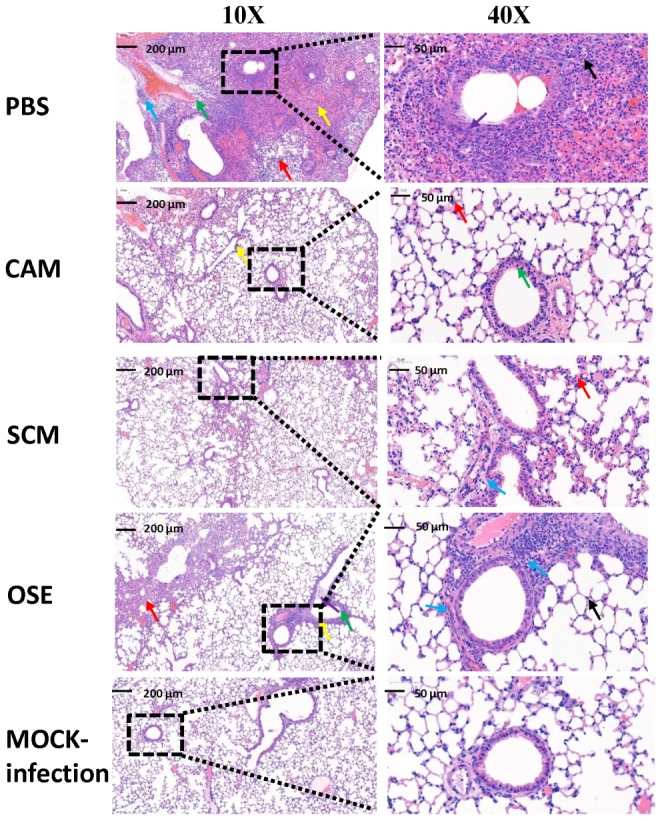
CAM and SCM effectively attenuated the lung pathology of A/4664T/2013 (H7N9) influenza virus-infected mice. The representative histopathologic changes of the lung tissues showed that less inflammatory infiltrate was observed in CAM- and SCM-treated groups compared with the PBS group. Lungs were fixed in 10% neutral-buffered formalin for 7 days and were cut into 4-μm-thick sections and stained with hem-atoxylin-eosin (H&E) staining. The digital images of whole-lung sections were captured by using a panoramic scanner slide scanner (3D HISTECH Pannoramic MIDI, Hungary). All micrographs were taken at ×10 or ×40 magnification. The inflammatory situations are symbolized by the arrow color. Black arrows represented neutrophils. Red arrows showed inflammatory cell infiltration and blue arrow showed more inflammatory cell infiltration. Thickening of alveolar wall (yellow arrow) and epithelial cell shedding (green arrow) were observed in the PBS group.

Subsequently, we examined the lung tissues of H7N9- or mock-infected mice treated or untreated with CAM or SCM for the histopathological changes. As shown in Figure 5, no signs of pathological changes were observed in the mock-infected (i.e., normal) mice. In the PBS treatment group, the structure of focal alveoli was destroyed, and alveolar walls were blurred. A large number of inflammatory cell infiltrates, including neutrophils (black arrows) and macrophages (red arrows), were seen. Meanwhile, exudation of protein-rich edema fluid (yellow arrows) was found in some alveolar spaces and bronchial lumens, and severe inflammatory cell infiltration (green arrow) was observed around the blood vessels. In the CAM treatment group, some inflammatory cell infiltrates (red arrows) were seen in the alveolar wall and around the blood vessels (yellow arrows). Bronchial epithelial cells were tightly arranged with clear boundaries. Thus, CAM treatment could effectively attenuate histopathological appearance and mitigate inflammatory responses. The histopathological changes of the lung tissues in mice treated with OSE were very similar to those in mice treated with CAM and SCM, in which only a few inflammatory cell infiltrates (blue arrows) were visible around the bronchi and blood vessels, and some loose cytoplasmic ribosomes in local bronchial epithelial cells were seen (yellow arrows). A qualitative histological assessment of the lung tissues was performed by two independent pathologists in a blinded manner. The mean cumulative histological scores in the CAM-, SCM-, OSE-treatment groups and the mock-infection group were significantly lower than that in PBS control group (Supplementary Table S1). The ratio of the alveolar/parenchymal area in the CAM-, SCM-, OSE-treatment groups and the mock-infection group were also remarkably lower than that in PBS control group (Supplementary Table S2). We also performed an immunohistochemical staining assay to quantitatively evaluate the expression of the viral NP in the lung tissues of mice infected with H7N9 influenza virus. We found that the integrated optical density (IOD) in the CAM-, SCM-, and OSE-treatment groups was significantly lower than that in PBS control group (Supplementary Figure S3 and Supplementary Table S3). All these results suggest that like OSE, CAM and SCM can effectively inhibit the influenza A H7N9 virus infection *in vivo* and attenuated the histopathological changes in the lungs of virus-infected mice.

### CAM and SCM Inhibited Influenza Virus Infection at an Early Step of Infection

**FIGURE 6:**
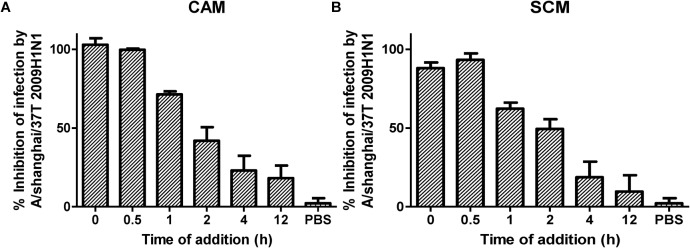
**(A,B)** Time of addition assay for analysis of mechanism of CAM and SCM against influenza A/shanghai/37T (2009H1N1) infection. MDCK cells cultured in DMEM supplemented with 10% FBS (Biowest, France), penicillin (100 U/ml), and streptomycin (10 μg/ml) at 37°C/5% CO_2_ were infected with influenza A/shanghai/37T (2009H1N1) at 0.1 MOI in DMEM containing 2 μg/mL TPCK-Trypsin. CAM and SCM diluted in DMEM to give a final concentration of 20 μM were added to mixture of virus and cells at 0, 0.5, 1, 2, 4, and 12 h post infection. Inhibition activity of CAM and SCM was measured by CPE reduction assay using CCK-8, according to the manufacturer’s instruction. The data were presented as means ± SE of triplicate assays and the experiment was repeated at least twice.

The influenza replication cycle consists of several steps. To elucidate the mechanism of action of CAM and SCM, a time-of-addition experiment was performed, as described previously (Droebner et al., 2017). CAM and SCM were added to MDCK cells at 0, 0.5, 1, 2, 4, and 12 h, respectively, after infection with A/Shanghai/37T/2009(H1N1) at MOI of 0.1. As shown in Figure 6, CAM and SCM exerted 99 and 93% inhibition on A/Shanghai/37T/2009(H1N1) infection, respectively, when added to cells at 0.5 h p.i., while their inhibitory activity was reduced to 23 and 19%, respectively, when they were added to cells at 4 h p.i. These results indicate that CAM and SCM inhibit influenza virus infection at an early step, possibly the viral entry stage.

### CAM and SCM Inhibited the Entry of H7N9 Pseudovirus Into MDCK Cells

**FIGURE 7:**
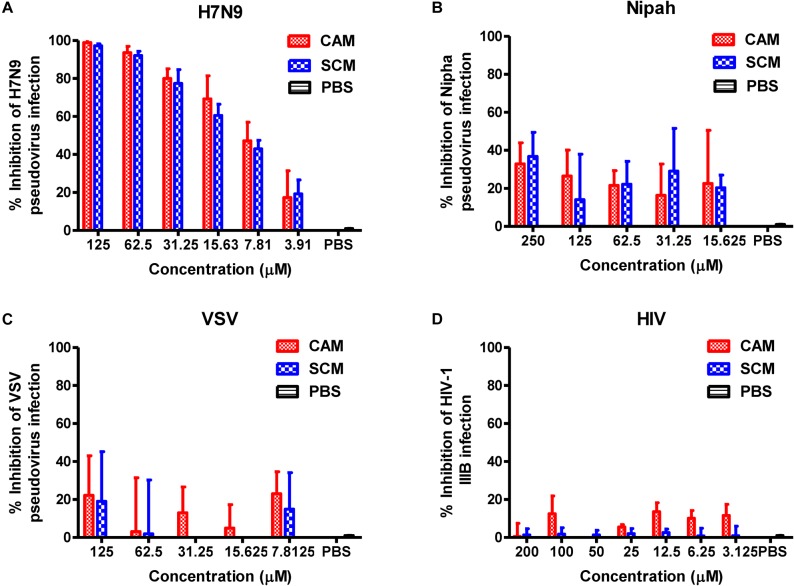
CAM and SCM inhibited the entry of H7N9 pseudovirus into MDCK cells. **(A)** CAM and SCM inhibited the entry of A/Shanghai/4664T/2013(H7N9) pseudovirus into MDCK cells. **(B)** CAM and SCM did not inhibit entry of Nipha pseudovirus into Huh7 cells. **(C)** CAM and SCM exhibited no inhibitory activity on VSV pseudovirus entry into U87 cells. **(D)** CAM and SCM did not inhibit HIV-1 IIIB infection in MT-2 cells. The data were presented as means ± SE of triplicate assays and the experiment was repeated at least twice.

To determine whether CAM and SCM could block the entry of the influenza virus into the target cells, we used the plasmid Env-H7N9-pNL4-3.luc.R-E- to construct pseudotyped H7N9 influenza virus particles, which express the two envelope proteins of the influenza A virus, HA and NA. The effect of CAM and SCM on the entry of this pseudotyped H7N9 IAV was detected by single-cycle pseudovirus infection assay. Pseudovirus expressing VSV G protein or Nipha virus (NiV) G protein was included as control. The result showed that CAM and SCM could effectively inhibit H7N9 pseudovirus infection in a dose-dependent manner with IC_50_ of 8.98 ± 3.93 and 10.85 ± 1.19 μM, respectively (Figure 7A), while CAM and SCM had no effect on infection by Nipha pseudovirus (Figure 7B) or VSV-G pseudovirus (Figure 7C), suggesting that antiviral ability might be related to HA or NA. Since these pseudoviruses contain the HIV-1 NL4-3 internal genes, we further investigated if these two compounds would have any effect on live HIV-1 IIIB infection. We found that neither CAM nor SCM had inhibitory effect on HIV-1 IIIB infection (Figure 7D). Collectively, these results indicate that both CAM and SCM are able to block the entry of influenza virus into the target cell, which represents the early step of virus replication.

### CAM and SCM Had No Effect on Virus Attachment to Host Cells or on Viral NA Activity

**FIGURE 8:**
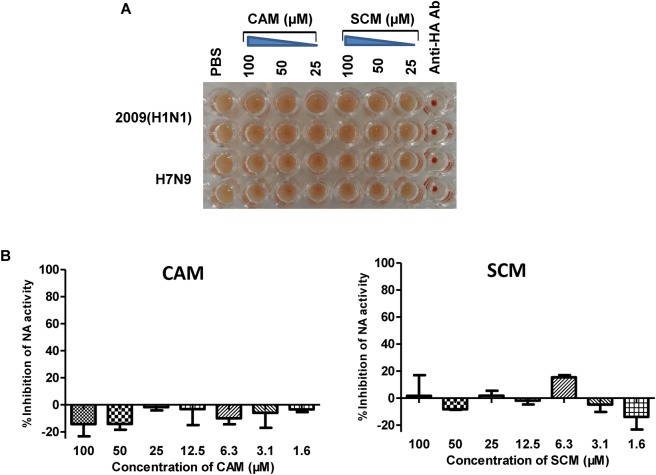
Haemagglutination inhibition (HI) assay and neuraminidase activity inhibition assay. **(A)** CAM and SCM did not block the attachment of influenza virus to chicken RBCs. Twenty-five μL of A/shanghai/37T/2009H1N1 and pseudoviruses of H7N9 (∼4 hemagglutination units) were mixed with an equal volume of CAM and SCM at 100, 50, and 25 μM, respectively, or anti-H1N1 antibody (10 μg/ml) as the positive control. After incubated at room temperature for 1 h, 50 μL of 1% (v/v) chicken RBCs were added, followed by an incubation at room temperature for 1 h. Haemagglutination was observed and record. **(B)** The inhibition of CAM and SCM on NA activity. The data were presented as means ± SE of triplicate assays and the experiment was repeated at least twice.

HA is a major membrane glycoprotein responsible for virus-cell receptor interaction and virus attachment to the target cell, the first step of viral entry (Fan et al., 2015), while NA is another membrane glycoprotein responsible for the release of the newly produced viral particles (NA activity). We performed a haemagglutination inhibition (HI) assay using chicken red blood cells (RBCs) to determine whether CAM or SCM could bind to HA on the virus surface to effectively inhibit the HI activity of HA. As shown in Figure 8, CAM and SCM at the concentration as high as 100 μM could not inhibit HA-mediated RBC agglutination (Figure 8A), nor could it inhibit NA activity (Figure 8B). These results suggest that CAM and SCM may not interfere with HA-mediated virus attachment and NA-mediated virus release, but other steps of viral entry.

## Discussion

In recent years, a number of avian influenza viruses, such as H7N9, H5N6, H10N8 and H9N2, have gained the ability to cross the species barrier to infect humans, posing a significant threat to public health worldwide (Chen et al., 2014; Bi et al., 2016; Zhu et al., 2016; Yao et al., 2018). Five major epidemic waves of H7N9 IAV infection have occurred since it first emerged in March 2013^2^. People infected with the H7N9 virus suffer from severe pneumonia and its complications, which can be fatal (Zhu et al., 2016). Although the NA inhibitor oseltamivir has been widely used to treat influenza virus infection, long-term oseltamivir treatment often leads to the emergence of drug-resistant viruses (Khodadad et al., 2015; Kiso et al., 2017). It has been reported that almost all seasonal influenza H1N1 viruses circulating in the United States in 2008–2009 were oseltamivir-resistant isolates (Kao et al., 2010). This calls for the development of new antiviral therapeutics against influenza viruses with resistance to the current antiviral drugs. Small-molecule compounds with broad-spectrum antiviral activity could offer a second line of defense when influenza epidemics or pandemics occur.

By screening an FDA-approved drug library, we identified two antihistamine drugs, CAM and SCM, which have had clinical application in the treatment of hay fever, rhinitis, urticaria, and asthma, with potent antiviral activity against avian influenza virus H7N9 with IC_50_ at low μM level. More importantly, both drugs could also inhibit infection by other influenza A viruses, such as 2009(H1N1), 1934(H1N1), and 1989(H3N2) strains, as well as an influenza B virus, B/Shanghai/2017(BY), suggesting that both CAM and SCM have broad antiviral activity against divergent influenza A strains and one influenza B strain. The result from an animal study showed that i.p. injection of CAM or SCM at 10 mg/kg/day for 5 days resulted in full protection of mice against challenge with lethal dose of avian influenza virus H7N9, indicating that these two antihistamine drugs have good potential for clinical use in the treatment, or, possibly, prevention of influenza virus infection.

The life cycle of the influenza virus can be divided into seven stages, as follows: (1) Attachment - HA, which can be cleaved into HA1 and HA2 subunits (Skehel, 2009), binds to sialic acid receptors on the host cell surface, facilitating viral entry into the cell (Yang et al., 2013). (2) Endocytosis - When virions are transported into the endocytic vesicle, the low pH environment (5.0–5.5) triggers the conformation of HA to mediate fusion of the viral and endosomal membranes (Xu and Wilson, 2011). These events are followed by (3) transcription, Translation, Replication, (4) packaging and (5) release of the newly produced viral particles out of the cell (Das et al., 2010; Lai et al., 2016). To determine which stage was targeted by CAM and SCM, we conducted time-of-addition and pseudovirus entry experiments. We found that both CAM and SCM target at the early stage of influenza virus life cycle by blocking entry of the influenza virus into the host cell. As described above, the entry stage consists of the early step of viral attachment to cells and late stage of viral endocytosis. Our study has shown that neither CAM nor SCM can block the attachment of 2009 H1N1 virus to chicken RBCs, suggesting that these two antihistamine drugs do not affect viral attachment to host cells, but may target the other steps of viral entry. More recently, Kouznetsova et al. (2014) and He et al. (2015) have reported that chlorcyclizine HCl (CCZ), an over-the-counter antihistamine drug for allergy symptoms, exhibits potent inhibitory activity against HCV infection by targeting viral entry into host cells, although the exact mechanism of action has not been clarified. Kouznetsova et al. (2014) and He et al. (2015) identified 53 compounds that block Ebola virus-like particle entry, three of which are antihistamine drugs, Clemastine, Maprotiline, and Benztropine. Similarly, however, they have not determined which stage of viral entry is targeted by these antihistamine drugs. Therefore, further studies on the mechanism(s) of action of these antihistamine drugs against viral infection are warranted. Histamine is a physiologically active substance that binds and activates histamine H1 and H2 receptors in the respiratory tract, brain, skin vasculature, and heart. Antihistamine drugs block the action of histamine, therefore reducing such symptoms as sneezing, rhinitis, rhinorrhea, erythema, pruritus, and urticaria. The first generation of antihistamines, for instance, CAM or SCM, have the ability to cross the blood-brain barrier and penetrate human central nervous cells to bind histamine receptors because of their anti-cholesterol properties. In the present study, we found that both CAM and SCM also possess the ability to inhibit influenza virus infection. Therefore, application of CAM or SCM for treatment of influenza may have complementary effect, i.e., inhibiting influenza virus infection and attenuating the inflammatory responses and allergic syndromes caused by viral infection. Furthermore, CAM or SCM may be use prophylactically in healthy individuals during an influenza epidemic or pandemic for prevention of influenza virus infection.

In summary, based on an FDA-approved drug library, we have identified two FDA-approved antihistamine drugs, CAM and SCM, that exhibit potent inhibitory activity against divergent influenza A strains and one influenza B strain and that protect mice from fatal challenge of avian H7N9 influenza virus. These antihistamine drugs inhibit influenza virus infection by targeting the early stage of virus life cycle, viral entry into the host cells. These results suggest that both CAM and SCM have potential for clinical use in the treatment and prevention of influenza virus infection.

## Author Contributions

SJ and LL conceived and designed the experiments. WX, SX, JP, PL, and QW performed the experiments. WX, LL, and SJ analyzed the data and wrote the manuscript.

## Conflict of Interest Statement

The authors declare that the research was conducted in the absence of any commercial or financial relationships that could be construed as a potential conflict of interest.

## Footnotes

1 http://www.who.int/csr/don/26-october-2017-ah7n9-china/en/

2 http://www.who.int/mediacentre/factsheets/fs211/en/
