# Supplementary material for: The Antihistamine Drugs Carbinoxamine Maleate and Chlorpheniramine Maleate Exhibit Potent Antiviral Activity Against a Broad Spectrum of Influenza Viruses
Source: Front Microbiol. 2018 Nov 6;9:2643. doi: 10.3389/fmicb.2018.02643 (PMC6232386; doi:10.3389/fmicb.2018.02643)
Supplement: Supplementary file 1 [file Data_Sheet_1.docx]

**Supplementary Material**

**The antihistamine drugs carbinoxamine maleate and chlorpheniramine maleate exhibit potent antiviral activity against a broad spectrum of influenza viruses**

Wei Xu^1^, Shuai Xia^1^, Jing Pu^1^, Qian Wang^1^, Peiyu Li^1^, Lu Lu^1*^, Shibo Jiang^1,2*^

^1^Shanghai Public Health Clinical Center & School of Basic Medical Sciences, Key Laboratory of Medical Molecular Virology of MOE/MOH, Fudan University, Shanghai, China

^2^Lindsley F. Kimball Research Institute, New York Blood Center, New York, NY, USA

***Correspondence:**

Dr. Shibo Jiang

shibojiang@fudan.edu.cn

Dr. Lu Lu

lul@fudan.edu.cn

**Table S1.** Histological assessment of lungs in influenza virus-infected mice treated with CAM, SCM and OSE.

| Group | alveolar congestion | hemorrhage | Neutrophilic infiltration | Hyaline membranes | Alveolar epithelium | Mean cumulative  Histological score |
| --- | --- | --- | --- | --- | --- | --- |
| PBS | 1.42±0.79 | 0.92±0.33 | 1.12±0.72 | 0.72±1.01 | 3.08±1.32 | 7.25±1.89 |
| Mock | 0.65±1.18* | 1.25±0.44 | 0.30±0.47* | 0.50±1.05 | 0.00±0.00* | 2.70±1.17** |
| CAM (1 mg/kg/day) | 0.10±0.31* | 2.85±0.67* | 0.60±0.50* | 0.00±0.00 | 2.1±1.97 | 5.65±2.85* |
| CAM (10 mg/kg/day) | 0.00±0.00* | 0.85±0.62 | 1.02±0.36 | 0.42±0.98 | 1.85±1.29 | 4.15±1.78** |
| SCM (1 mg/kg/day) | 0.05±0.22* | 1.08±0.42 | 0.80±0.61 | 0.05±0.22 | 2.60±0.22 | 4.57±1.87** |
| SCM (10 mg/kg/day) | 0.00±0.00* | 0.65±0.49 | 0.30±0.47* | 0.00±0.00 | 1.40±1.05 | 2.35±1.35** |
| OSE (1 mg/kg/day) | 0.70±1.22* | 0.90±0.31 | 0.70±0.47 | 0.40±0.50 | 2.15±1.04 | 4.85±0.88** |

*, ** Represent significant difference at p<0.05 and p<0.005, respectively, between each treatment group and the PBS group, analyzed with the One-way ANOVA analysis using GraphPad Prism software.

**Table S2.** Analysis of ratio of the alveolar/parenchymal area in lungs of influenza virus-infected mice treated with CAM, SCM and OSE.

| Group | Ratio of the alveolar/parenchymal area (%) |
| --- | --- |
| PBS | 64.71 |
| Mock | ND |
| CAM (1mg/kg/day) | 26.86 |
| CAM (10mg/kg/day) | ND |
| SCM (1mg/kg/day) | 11.90 |
| SCM (10mg/kg/day) | ND |
| OSE (1mg/kg/day) | 3.88 |

ND, not detect

**Table S3.** Analysis of integrated optical density

| Group | Integrated optical density (IOD) | P value* |
| --- | --- | --- |
| PBS | 75,638 |  |
| CAM (1 mg/kg/day) | 2,844 | <0.05 |
| CAM (10 mg/kg/day) | 2,223 | <0.05 |
| SCM (1 mg/kg/day) | 298 | <0.05 |
| SCM (10 mg/kg/day) | 1,518 | <0.05 |
| OSE (1 mg/kg/day) | 4,623 | <0.05 |

* Represent significant difference between each treatment group and the PBS group, analyzed with the unpaired *t* test using GraphPad Prism software.

**Table S4.** Inhibitory activity of CAM and SCM agaisnt infection by 2009 H1N1 and H3N2 strains detected with plaque reduction assay

| Compound | IC_50_ (µM) | |
| --- | --- | --- |
|  | 2009 H1N1 | H3N2 |
| CAM | 14.49 ± 4.80 | 2.71 ± 0.36 |
| SCM | 13.32 ± 4.67 | 3.76 ± 0.98 |

Data were obtained from two independent experiments and presented as the mean ± standard deviation.


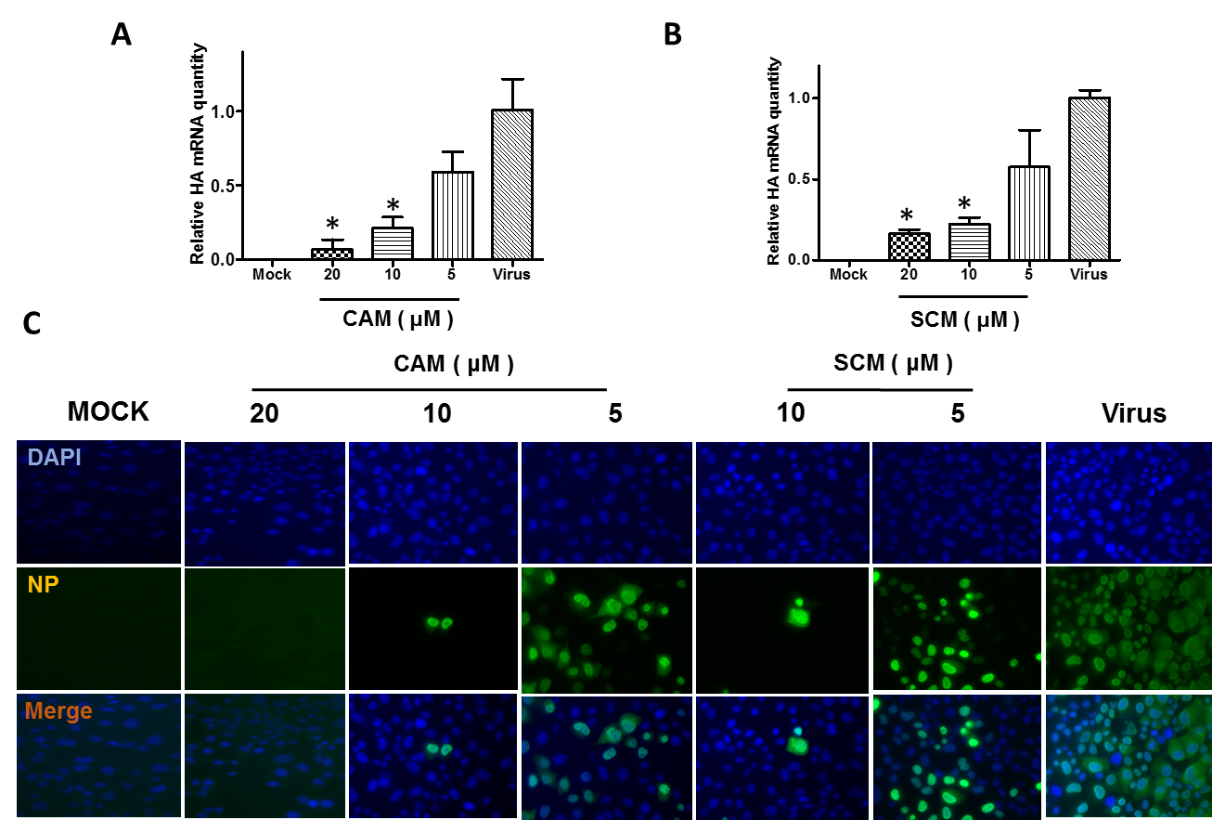


**Figure S1.** Inhibition of CAM and SCM on influenza A/PR/8/1934 (H1N1) infection detected with different methods. MDCK cells were infected with 100 TCID_50_ influenza A/PR/8/1934 (H1N1) in the absence or presence of CAM and SCM at the indicated concentration for 1 h and then the medium was replaced with fresh DMEM. After incubation at 37^o^C for 12 h, the mRNA level of the viral HA gene in MDCK cells treated with CAM (A) or SCM (B) was measured by RT-PCR. (C) Expression of viral NP on the influenza virus-infected cells treated with CAM or SCM was detected by the indirect immunofluorescence assay. The cells were stained with an anti-NP monoclonal antibody for 3 h and then with the goat anti-mouse IgG (H+L) antibody Alexa Fluor 488 for 1 h. Nuclei were stained by DAPI.


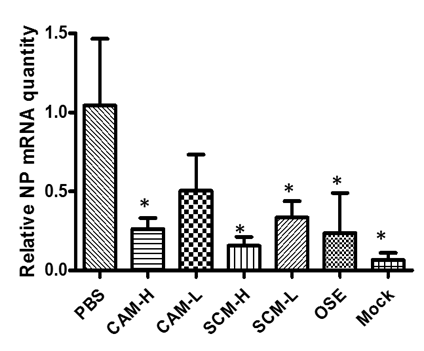


**Figure S2**. Inhibition of CAM and SCM on *in vivo* influenza A/4664T/2013 (H7N9) infection in mice. Mouse lung was immersed in TRIzol™ reagent and homogenized. Total RNA were determined by two-step RT-qPCR. Relative changes were normalized to the GAPDH gene and quantification was calculated by the 2-△△CT method. * indicates significant differences (P<0.05) between treated group and the PBS group.

**
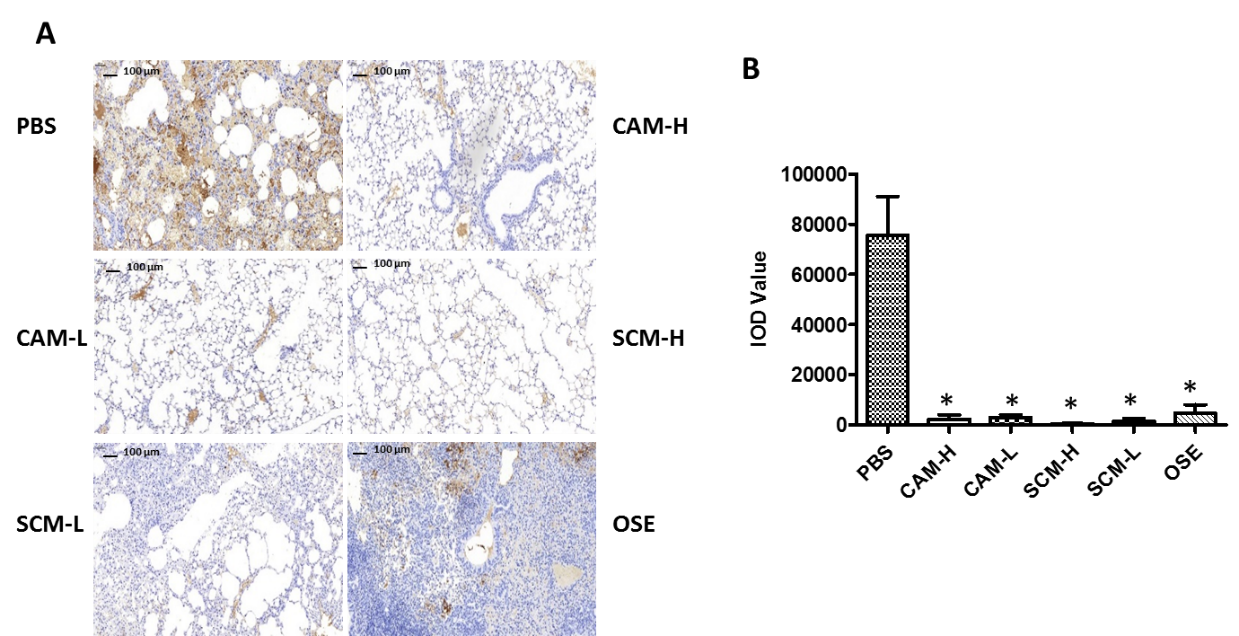
**

**Figure S3.** Histopathological changes of lung tissues in mice infected by influenza virus A/4664T/2013 (H7N9) and treated with CAM or SCM. The right lung were fixed in 10% buffered formalin solution and embedded in paraffin. The tissues were cut into 4 μm sections and immunohistochemically stained with the primary antibody against influenza A virus' NP and the goat anti-mouse IgG (H+L) highly cross-adsorbed secondary antibody sequentially. The integrated optical density (IOD) value, which is considered as the relative expressions of NP, was determined from three randomly selected views and captured under an optical microscope (×200), and analyzed with the Image-Pro Plus 6.0 software (Media Cybernetics, Inc., Rockville, MD, USA). Scale Bar: 100 μm. (A) Immunohistochemical staining for NP of influenza virus A H7N9 in mice lungs; (B) Quantitative data for immunohistochemical staining. The IOD values were determined as described above.


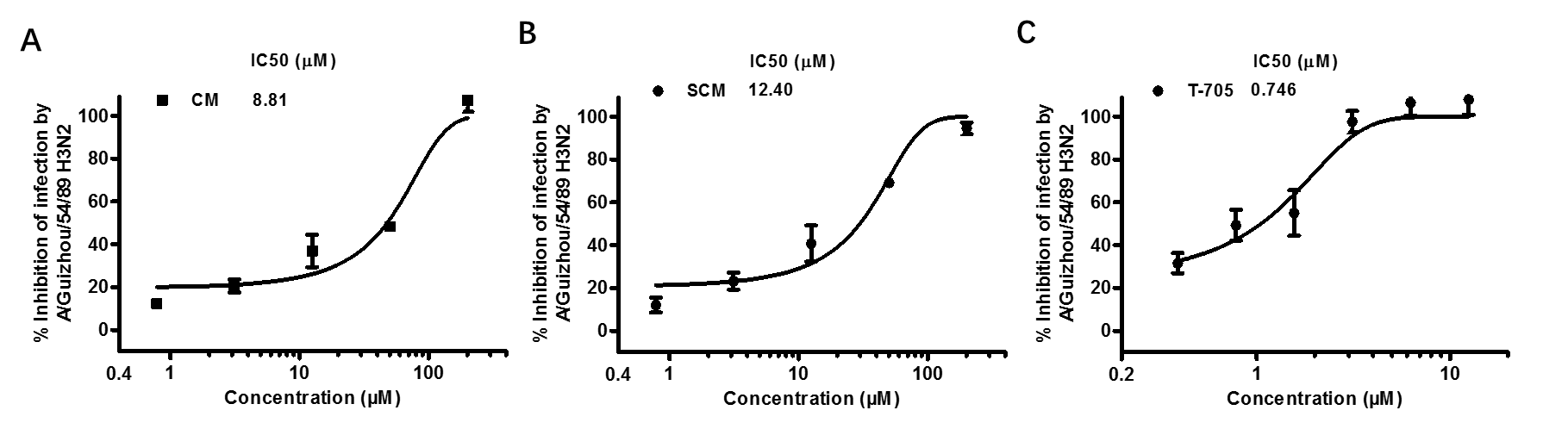


**Figure S4**. Inhibition of CAM and SCM on influenza virus infection *in vitro*. The IC_50_ values of (A) CAM, (B) SCM and (C) Favipiravir (T-705) were measured for their inhibition of infection by A/Guizhou/54/1989(H3N2) in MDCK cells at 100 TCID_50_ in the presence of increasing concentrations of the compounds. The inhibitory activity of CAM and SCM was determined at 72 h post infection by CPE reduction assay using CCK8. Data are expressed as mean ± standard error (SE) of triplicate assays.
